# Venous thromboembolism prophylaxis may cause more harm than benefit: an evidence-based analysis of Canadian and international guidelines

**DOI:** 10.1186/s12959-018-0180-6

**Published:** 2018-10-10

**Authors:** Andrew Kotaska

**Affiliations:** 1 Women’s & Children’s Health, Northwest Territories Health and Social Services Authority, Stanton Territorial Hospital, Yellowknife, NT X1A 2N1 Canada; 2 School of Population and Public Health, University of British Columbia, Vancouver, Canada; 3 Department of Obstetrics and Gynaecology, University of Manitoba, Winnipeg, Canada; 4 Department of Obstetrics and Gynaecology, University of Toronto, Toronto, Canada

**Keywords:** Venous thromboembolism, Prophylaxis, Guidelines, Evidence-based medicine, Conflict of interest, Deep vein thrombosis, Pulmonary embolism

## Abstract

A majority of deep vein thromboses identified in screening studies of hospitalized patients remain clinically insignificant. Guidelines based on these studies markedly overestimate the risk of clinical venous thromboembolism (VTE) and the benefit of heparin prophylaxis. Accordingly, in 2012, the American College of Chest Physicians (ACCP) removed screening studies from the 9th edition of its Antithrombotic and Thrombolytic Therapy guideline (AT9), and downgraded recommendations. Involvement of authors of the 8th edition (AT8) was restricted due to financial and intellectual conflicts of interest. However, the first author of AT8 subsequently wrote a “Getting Started Kit,” widely distributed to help Canadian hospitals develop VTE protocols. Based on screening studies reporting *asymptomatic* VTE, it lacks estimates of the magnitudes of benefit or harm from low molecular weight heparin (LMWH), yet advises prophylaxis in almost all hospitalized patients. Most Canadian hospitals have implemented guidelines based on this kit. Guidelines from the U. K National Institute for Health and Care Excellence and the U.S. Agency for Healthcare Research and Quality recommend a similar approach. However, a critical review of evidence reveals that most hospitalized patients have a risk of *clinical* VTE equal to or lower than the bleeding risk from LMWH. Most hospitalized patients should not receive LMWH until and unless randomized trials show more benefit than harm. Guidelines recommending liberal LMWH prophylaxis in hospitalized patients are not evidence based and should be critically re-examined.

**Electronic supplementary material:**

The online version of this article (10.1186/s12959-018-0180-6) contains supplementary material, which is available to authorized users.

## Background

Venous thromboembolism (VTE) is an important clinical concern in medical and surgical patients. Up to one third of VTE are pulmonary emboli (PE), which can be rapidly fatal in up to 10% of cases; and severe post-thrombotic syndrome occurs in approximately 10% of patients after symptomatic deep vein thrombosis (DVT) [1]. High-risk patients are targeted for prophylaxis; however, myriad associated clinical factors make it difficult to identify individual patients destined to experience VTE. Scoring systems designed to identify such patients have been implemented without validation in randomized trials.

The American College of Chest Physicians (ACCP) has published an extensive series of Antithrombotic and Thrombolytic Therapy guidelines. Until and including the 8th edition (AT8), these guidelines were based on studies that screened patients for asymptomatic DVT [2]. However, few asymptomatic DVT become clinically significant, making it a poor surrogate for clinically important disease. Accordingly, the evidence was thoroughly re-evaluated in the ninth edition (AT9), published in 2012. Evidence previously rated as high quality is now moderate, and evidence previously rated as moderate quality is now low. To a large extent, strong recommendations of AT8 have been replaced by weak recommendations in AT9 [3, 4]. Specifically:

- It is acknowledged that the use of asymptomatic, screening-detected thrombosis as an outcome substantially over-estimates the clinical benefit of prophylaxis.
- Clinically evident VTE rather than asymptomatic VTE is now used for estimates of VTE incidence and calculations of prophylaxis benefit.
- The financial and intellectual conflicts of interest of leading experts and prior authors were felt to be “highly problematic,” so their involvement was restricted.

For general surgical patients, scoring systems are still advised to estimate the risk of VTE; however, bleeding risk is now acknowledged by a recommendation that the post-operative incidence of clinical VTE should exceed 3% to warrant chemo-prophylaxis [5–7]. After major orthopedic surgery, lower potency prophylaxis has been found to be effective and cause fewer wound and joint complications [8–11]. In medical patients, large randomized trials of LMWH prophylaxis demonstrate little or no benefit, calling into question the utility of poorly validated tools used to estimate risk [12–14].

This re-evaluation of the evidence and downgrading of recommendations has not been translated into corresponding changes on the front lines of clinical practice. Six years later, Canadian and international hospital VTE guidelines remain based on outdated evidence from AT8. This paper critically reviews those recommendations using an evidence-based lens and explores the role of conflict of interest in their generation and dissemination.

## Main text

### Accreditation Canada’s required organizational practice

Accreditation Canada is a national organization that sets hospital safety standards. Required Organizational Practices (ROPs) are deemed critical to safety, and hospitals must comply or lose their accreditation. In 2011, Accreditation Canada instituted a ROP requiring hospitals identify and provide prophylaxis to admitted adult patients at elevated risk of VTE. Given a lack of clarity regarding which patients benefit from prophylaxis, Canadian hospitals needed direction. Accordingly, a “Getting Started Kit” was developed by “Safer Healthcare Now!” a self-described “flagship program of the Canadian Patient Safety Institute and a national program supporting Canadian healthcare organizations to improve safety through the use of quality improvement methods and the integration of evidence in practice” [15]. A “free resource designed to help (hospitals) implement interventions in (their) organization … the Getting Started Kit contains clinical information, information on the science of improvement, and everything (hospitals) need to know to start using the intervention” [16, 17].

The Getting Started Kit has the same first author as AT8. Also based on studies that screened for asymptomatic DVT, it reports incidences of 10–40% for medical patients and 15–80% for surgical and trauma patients (Table 1). To an experienced clinician, these numbers are strikingly discordant from clinical practice. Although the authors state: “(Table 1) lists the DVT incidence for various hospitalized patient groups if no prophylaxis is given and screening for asymptomatic DVT is performed,” they conclude **“**based on the significant, known rates of VTE as well as its acute and long-term consequences, it can be seen that nearly every hospitalized patient should receive thromboprophylaxis” [15].

**Table 1:** Asymptomatic VTE risk from screening studies (from the VTE Getting Started Kit, Patient Safety Institute of Canada; open source)

Patient Group	DVT Incidence (%)
Medical patients	10-26
Major gynecologic, urologic, or general surgery	15-40
Neurosurgery	15-40
Tibial fracture	20-40
Congestive heart failure	20-40
Stroke	11-75
Knee/hip arthroplasty	40-60
Hip fracture	40-60
Major trauma	40-80
Spinal cord injury	60-80
Critical care patients	15-80

This conclusion is highly misleading. Few patients with *asymptomatic* DVT develop clinical VTE, and the incidence of clinical DVT is an order of magnitude lower than the incidence of asymptomatic DVT [18, 19]. In the large meta-analysis of general surgical patients referenced in AT9 (*n* = 5400), the baseline risk of clinical VTE without heparin was 0.89% [20]. The pooled risk of symptomatic DVT in another large meta-analysis of mixed surgical patients was 0.6% [21]. In a retrospective cohort study used to validate a surgical risk scoring system (*N* = 8216), the baseline risk was 0.28% for moderate risk and 0.9% for high-risk patients [5]. The incidence of symptomatic VTE in surgical patients is less than one tenth that of asymptomatic DVT. In randomized studies of LMWH in more than 25,000 medical and stroke patients, the incidence of symptomatic DVT and pulmonary embolus with placebo were less than 1% each, and large randomized trials have shown no net benefit of LMWH prophylaxis [13, 14, 22].

**Fig. 1:**
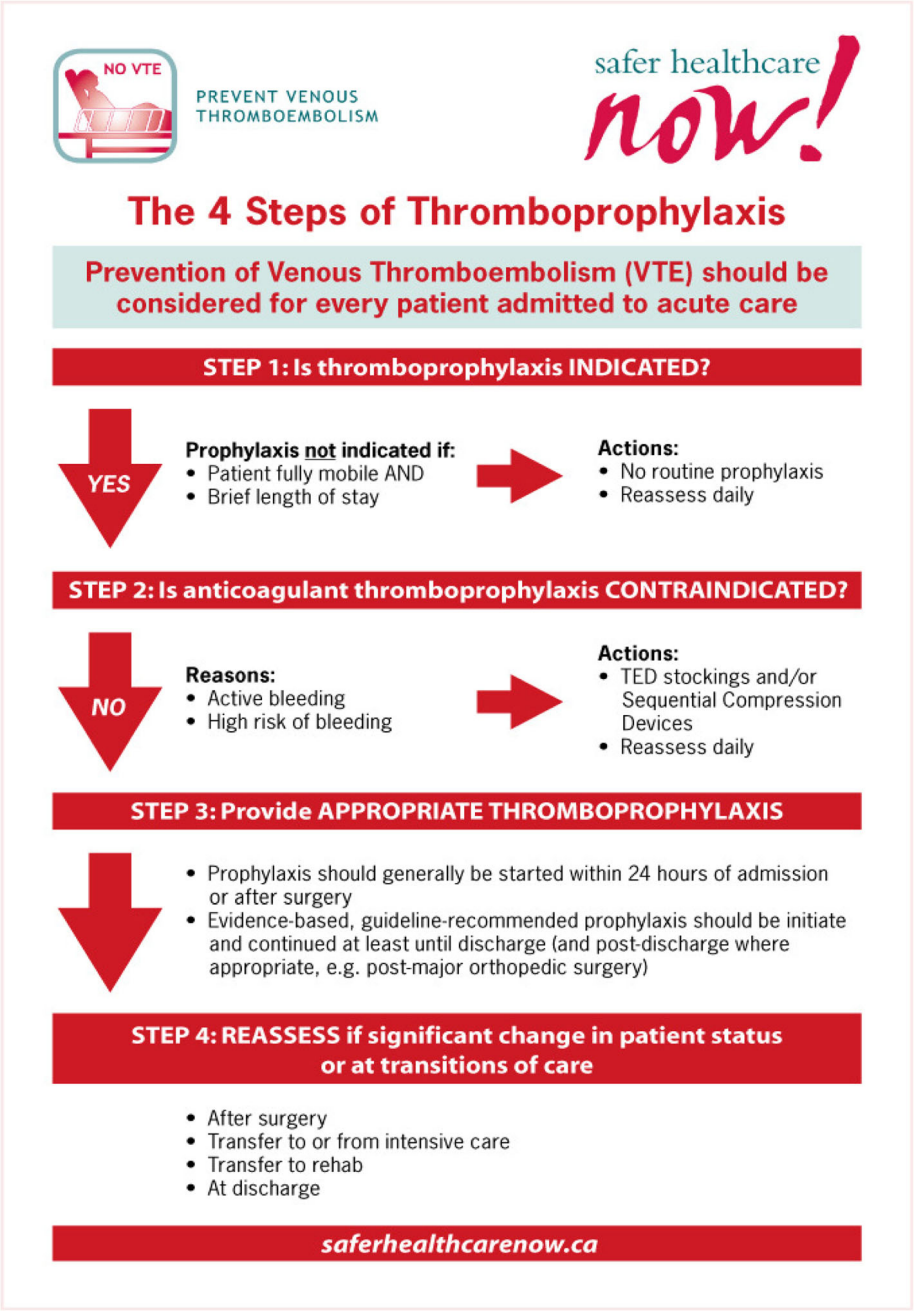
Preferred 'opt-out' decision tree for VTE prophylaxis. (from the VTE Getting Started Kit, Patient Safety Institute of Canada; open source)

However, none of this is mentioned in the Getting Started Kit. Alarmingly, the preferred thromboprophylaxis decision tree is one of ‘opt out’ (Fig. 1). Except for patients actively bleeding or at high risk of bleeding, it advises all patients receive LMWH unless fully mobile and admitted for less than 2 days. Without providing data, bleeding risk is dismissed: “Abundant data from meta-analyses and blinded, placebo controlled randomized trials have shown that clinically important bleeding secondary to prophylaxis with LDUH or LMWH is a rare event” [15]. In fact, LMWH prophylaxis causes significant increases in hemorrhage, which for many hospitalized patients likely equals or exceeds the risk of VTE prevented.

### VTE incidence and bleeding risk with LMWH after surgery

The Caprini Scoring system is used to identify patients at increased postoperative risk of VTE [6]. Its scoring sheet declares *asymptomatic* VTE incidences of 10 to 80% from screening studies and recommends chemoprophylaxis in patients with a score of 2 (“moderate risk”), or greater. The Caprini score has been shown to predict which patients will experience VTE; however, in the largest validation study, the risk of symptomatic VTE was less than 1% in “moderate” and “high-risk” general, vascular and urological surgery patients [5]. A majority of otolaryngology, gynecological, and plastic surgery patients also have a risk of symptomatic VTE under 1% - a magnitude of risk substantially lower than the 3% threshold felt necessary to warrant chemoprophylaxis in AT9 [23–25]. Even in “highest-risk” general surgical patients, the VTE risk was only 2% [5]. In contrast, almost all patients requiring surgical intensive care have a VTE risk above 3%, justifying chemo-prophylaxis [26].

For a risk scoring tool to be practical, it must be simple [27]. The Caprini score has 35 risk factors and is unwieldy to administer. A simpler risk-scoring tool is very promising [28]. Recognizing that the risk of hemorrhage from LMWH is not insignificant, its authors advise that future research should provide “data on the risk-stratified response to prophylaxis side by side with the risk stratified data on bleeding complications.” The meta-analysis of randomized trials referenced in AT9 included 5400 general surgical patients given LMWH or placebo [20]. Compared with placebo, LMWH reduced the absolute risk of clinical VTE by 0.68%, yielding a number needed to treat (NNT) of 147. However, LMWH increased major hemorrhages and hemorrhage requiring transfusion by absolute risk increases (ARI) of 1.5% and 3.8%, yielding numbers needed to harm (NNH) of 67 and 26 – lower than the NNT. More patients experienced bleeding caused by LMWH than avoided VTE: for every VTE prevented, two patients experienced major hemorrhage and seven received a transfusion. This data supports the AT9 recommendation that post-operative VTE risk should be at least 3% to justify LMWH [7].

The skepticism of orthopedic surgeons towards high-potency thromboprophylaxis is noteworthy. Increased wound and joint complications prompted early critical review of the evidence and more cautious recommendations [8–10]. Shorter, lower potency anticoagulation is now considered adequate after total joint replacement, with ASA 81 mg daily found to be non-inferior to rivaroxaban beyond the first 5 days [11].

### VTE and bleeding risk with LMWH in medical patients

AT9 recommends LMWH in acutely ill hospitalized medical patients according to the Padua Prediction Score [29]. This risk assessment tool divides patients into low and high-risk groups based on 11 risk factors. It’s validation study demonstrated clinical VTE in 11% of patients with a score of 4 or greater and 0.3% in patients with a score of less than 4 – a remarkable hazard ratio of 32 for a complex phenomenon [12]. Forty percent of all patients were deemed to be “high risk.” Ninety-seven percent of those who developed VTE had at least one of four common major risk factors: prior history of VTE; active cancer; known thrombophilia; or bed rest for at least three days.

The study was not randomized and clinicians were not aware of their patients’ VTE risk assessments. Administration of prophylaxis was left to clinical judgment. Fewer than 40% of high-risk patients received adequate thromboprophylaxis. The authors state that “randomization would have been unethical;” yet had patients been randomized, a full 50% would have received LMWH. They conclude “the lack of randomization of high-risk patients to receive thromboprophylaxis or not precludes a correct comparison between the two study groups … The Padua Prediction Score’s validity requires proper confirmation and validation from other large prospective studies.” Further validation studies have not been published.

The Padua prediction score provides modest observational evidence that medical patients with four recognized ‘very-high’ risk factors should receive LMWH. However, these factors were present in a minority of patients and the proportion may be lower in a general hospital setting. Much larger randomized trials demonstrate a baseline risk of VTE of 1% or less in general medical patients, and little or no impact of LMWH on the incidence of clinical VTE [13, 14, 22]. This is similar to the magnitude of risk after outpatient knee arthroscopy, for which LMWH is not recommended [30]. Excess risk of serious bleeding in medical patients is up to 0.5% [14]. Neither the Padua prediction tool nor randomized trials support liberal VTE protocols for medical patients or the conclusion that “nearly every hospitalized patient should receive thrombo-prophylaxis” [15].

### VTE and bleeding risk with postpartum LMWH

Evidence for LMWH prophylaxis in postpartum women is lacking [31]. Despite efforts to base AT9 on studies of *clinical* rather than *asymptomatic* VTE, the obstetrical portion was overlooked [32]. Drawn from a decision analysis based on screening studies, estimates of DVT risk after cesarean section (CS) are ten-fold higher than the incidence of *clinical* DVT [33]. Postpartum risk is not adjusted for the short period during which LMWH is administered, and the risks of LMWH have been overlooked. LMWH is recommended in women with common risk factors in whom the risk of clinical VTE is less than 0.1% during the first week postpartum. Giving LMWH for one week after typical CS, the NNT to prevent one VTE is 4000 [31, 34]. Approximately 1% of obstetrical VTE are fatal PE, yielding a NNT to prevent one PE-death of 400,000 [35].

Obstetrical organizations from the UK, Canada, Sweden, Australia and New Zealand have developed unvalidated guidelines based on risk factors taken from case-control studies, with little attention to the magnitude of risk [36–39]. Estimates of absolute risk reduction (ARR), NNT, ARI, and NNH are lacking. Except for women with a prior history of VTE or known thrombophilia, there is not observational or experimental evidence that LMWH prophylaxis reduces VTE after CS, even in ‘high-risk’ women [40–43].

However, LMWH after CS is associated with increased wound separation and re-hospitalization for wound complications, with ARIs of 3.8% and 1.3% respectively (NNH = 26 and 77) [43]. Since the NNT to prevent one VTE after typical CS is approximately 4000, some 50 women may experience wound complications from LMWH for every VTE prevented. The risk of severe hemorrhage from LMWH in postpartum patients is unknown. After CS, AT9 suggests an additional 2% risk of “major bleed” defined as “leading to death, transfusion, reoperation, or discontinuation of (heparin) therapy” [32]. In reality, birthing women are younger and healthier than most surgical patients, so the risk is likely lower. However, if the risk were only one tenth the ACCP estimate (0.2%), the NNH would be 500, and approximately eight women would experience serious hemorrhage from LMWH for each VTE prevented [30].

### Canadian and international hospital VTE guidelines

In response to Accreditation Canada’s ROP, most Canadian hospitals implemented VTE guidelines based on the Getting Started Kit. From a convenience sample of VTE protocols, procedures, and order sheets from 12 hospitals from 9 Provinces and Territories, all except one recommend liberal LMWH for most admitted hospitalized patients (Additional file 1). Similar to the Getting Started Kit, many recommend LMWH for all patients with an ‘opt out’ for very limited exclusions (Additional file 2). The remainder have adopted unvalidated risk scoring systems containing dozens of clinical factors, with a low threshold for treatment (Additional file 3). All except one lack estimates of the magnitude of benefit or harm that patients might experience from LMWH prophylaxis.

VTE guidelines for hospitalized patients from the U.K. National Institute for Health and Care Excellence (NICE) recommend a similar approach [44]. For medical and surgical patients, a link to a UK Dept. of Health VTE risk assessment tool is provided: “Any tick for thrombosis risk should prompt thromboprophylaxis according to NICE guidance … (unless) bleeding risk is sufficient to preclude pharmacological intervention” [45]. For obstetrical patients, the unvalidated RCOG guideline is recommended [31].

The U.S. Agency for Healthcare Research and Quality (AHRQ) guideline: Preventing Hospital-Acquired Venous Thromboembolism is based on AT8. The 2008 first edition is similar to the Getting Started Kit [46]. The 2nd edition (2016) continues to recommend “… the most widely used qualitative model in the United States, the University of California San Diego model … derived directly from tables in the AT8 guideline” [47]. All patients qualify for heparin unless they are fully mobile and remain in hospital for less than 48 h. Compared with more complicated, unpopular, individualized point-scoring systems, “this risk assessment model was considered intuitive and easy to use.”

### Conflicts of interest

Early ACCP guidelines fueled worldwide enthusiasm for VTE prevention and led to recommendations for liberal LMWH prophylaxis in hospitalized patients. In AT8 and guidelines based upon it, most hospitalized patients qualified for LMWH [2, 15, 45, 46]. The first author of AT8 disclosed “that he received grant monies from the Canadian Institute for Health Research, Sanofi-Aventis, and Pfizer … consultant fees from Bayer, Eisai, Glaxo Smith Kline, Lilly, Merck, Pfizer, Roche, and Sanofi-Aventis, along with speaker’s honoraria from Bayer, Calea, Oryx, Pfizer, and Sanofi-Aventis” [2]. Sanofi-Aventis, Pfizer, Bayer, GlaxoSmithKline, Lilly, and Merck produce (d) the anticoagulants enoxaparin, dalteparin, rivaroxaban, nadroparin, fondaparinux, and unfractionated heparin.

This author’s involvement was restricted from AT9 because of financial and intellectual conflicts of interest; however, he is the first author of the Getting Started Kit and remains the primary consultant for Accreditation Canada regarding VTE. Published three months after AT9, the Getting Started Kit presents asymptomatic screening data that exaggerate the benefit of LMWH. However, it references both AT8 and AT9, indicating that the author was aware of the widely accepted conclusion that most asymptomatic DVT are clinically irrelevant. This conclusion is not mentioned in the Getting Started Kit. The Kit was partly funded by an “unrestricted educational grant from Pfizer.” It does not contain a conflict of interest declaration.

Conflicts of interest have plagued guidelines for years [48]. With AT9, the ACCP made an unprecedented effort to address conflicts of interest, almost completely replacing authorship [4]. The presence of a conflict of interest does not necessarily mean that authors’ conclusions are biased; however, transparent disclosure allows editors, guideline committees, clinicians, and patients to evaluate potential bias and adjust their decisions accordingly. Striking differences in the recommendations of AT8 and AT9 parallel a striking difference in authors’ conflicts of interest. Six of seven authors of AT8 declared financial relationships to multiple companies that produce antithrombotic drugs. In contrast, one of five authors of AT9 declared any financial relationship.

Problems arise “not only from (authors’) financial but equally or perhaps more importantly, their intellectual conflict of interest” [4]. In practitioners’ and researchers’ enthusiasm to help patients, there is a tendency to believe that our recommendations and actions are beneficial. When evidence calls previous conclusions into question, objective re-evaluation may be difficult, perhaps more so when research and commercial consulting careers are involved.

### Evidence-based medicine

Enthusiasm for new cures is an essential stimulus for innovation in medicine and has driven VTE guidelines. However, many new therapies adopted without adequate evaluation have later been found to lack benefit or even harm patients. Although all hospitalized patients are at risk of clinical VTE, for most, the magnitude of risk and our ability to prevent it have been exaggerated. Asymptomatic DVT is not a meaningful surrogate outcome for clinical VTE, and the risk of LMWH has been overlooked.

Forty years ago, Archie Cochrane challenged the medical profession to be critical of new treatments and to carefully evaluate them before widespread adoption [49]. Evidence-based medicine was our collective response [50]. Evidence-based medicine intends to balance high-quality evidence with patient values and clinical expertise to achieve optimal outcomes [51]. Critical to this effort is estimation of the absolute magnitudes of benefit and harm: the ARR, ARI, NNT, and NNH for the prevention of VTE with LMWH in medical, surgical, and postpartum patients.

Randomized controlled trials (RCTs) are the accepted gold standard for measuring benefits and harms from medical therapy. Given that the incidence of clinical VTE in most hospitalized patients is small, trials must be large to have the power to detect benefit from LMWH. The logistics are daunting; however, the imperative is great. For a majority of hospitalized patients, a low baseline risk of VTE means a greater likelihood that harm from LMWH will outweigh benefit. A large NNT also means high cost for little benefit. For these reasons, Dr. Cochrane advised that therapies’ benefit be proven in adequately powered RCTs *before* their dissemination [52].

## Conclusion

There is moderate evidence that patients with a prior VTE, potent thrombophilia, active cancer, prolonged bedrest, major orthopedic or abdominal-pelvic surgery, or ICU admission should receive chemoprophylaxis in hospital and for several weeks afterwards [40, 53]. For these patients, ongoing research may continue to customize the potency and duration for individual circumstances [11]. However, a majority of hospitalized medical, surgical, and postpartum patients lack these risk factors. Their risk of VTE has been exaggerated in most Canadian and major international hospital guidelines.

Admittedly, reliable estimates of VTE risk in patients with multiple medical and surgical risk factors are lacking and clinical judgment is required. However, multiple risk factors from case-control studies do not multiply VTE risk exponentially, and the absolute magnitudes of benefit and harm from LMWH must be considered [4, 54]. Low thresholds for LMWH prophylaxis may cause more harm than benefit, and the assumption that every hospitalized patient should receive LMWH unless fully mobile is oversimplified and unjustified. Protocols that recommend LMWH for surgical patients with modest risk factors such as age over 60, BMI over 30, a respiratory condition, or surgery lasting more than an hour are not evidence-based and likely cause more harm than benefit.

Pannucci and colleagues’ advice that future research should provide “data on the risk-stratified response to prophylaxis side by side with the risk stratified data on bleeding complications” is a call to measure the NNT and NNH. This will particularly benefit the majority of hospitalized patients who are at modest risk of symptomatic VTE, for whom harm from LMWH may equal or exceed benefit. Only by balancing the NNT with the NNH can “physicians … make informed decisions about the risks and benefits of prophylaxis for individual patients” [28]. Randomized trials provide direct measurement of the magnitudes of benefit and harm from LMWH and are needed to validate scoring systems for most surgical and medical patients.

Rarely, a new therapy provides such clear benefit that dissemination is justified before thorough evaluation. That is not the case here. Guidelines based on the prevention of *asymptomatic* rather than *clinical* VTE exaggerate the benefits of LMWH therapy and obscure its harms. The widespread treatment of a majority of hospitalized patients with LMWH constitutes a massive experiment: without a power calculation, ethics review, measurement of benefit and harm, or informed patient consent. In light of the advances in scientific understanding of AT9, guidelines advising liberal LMWH prophylaxis should be critically re-evaluated using the tools of evidence-based medicine. Until net benefit is proven, most hospitalized medical, surgical, and obstetrical patients should not receive LMWH prophylaxis except in the context of randomized trials.

## Additional files

Additional file 1: Selective hospital VTE screening tool: treats a minority of hospitalized patients. (PDF 127 kb)

Additional file 2: 'Opt-out' hospital VTE screening tool: treats almost all hospitalized patients. (PDF 252 kb)

Additional file 3: Complex liberal hospital VTE risk-scoring tool: treats most hospitalized patients. (PDF 130 kb)

## Abbreviations

ACCP: American College of Chest Physicians
AHQR: Agency for Healthcare Research and Quality
ARI: Absolute risk increase
ARR: Absolute risk reduction
CS: Caesarean scetion
DVT: Deep vein thrombosis
LMWH: Low molecular weight heparin
NICE: National Institute for Health and Care Excellence
NNH: Number needed to harm
NNT: Number needed to treat
PE: Pulmonary embolism
RCT: Randomized controlled trial
ROP: Required organizational practice
VTE: Venous thromboembolism
