# Supplementary material for: Venous thromboembolism prophylaxis may cause more harm than benefit: an evidence-based analysis of Canadian and international guidelines
Source: Thromb J. 2018 Oct 10;16:25. doi: 10.1186/s12959-018-0180-6 (PMC6178253; doi:10.1186/s12959-018-0180-6)
Supplement: Supplementary file 1 — Selective hospital VTE screening tool: treats a minority of hospitalized patients. (PDF 127 kb) [file 12959_2018_180_MOESM1_ESM.pdf]

CANADIAN COMMUNITY HOSPITAL

(Patient ID)

VENOUS THROMBOEMBOLISM RISK ASSESSMENT

ADMISSION RISK ASSESSMENT

☐ Yes ☐ No: History of VTE or known thrombophilia

☐ Yes ☐ No: Planned hip or knee arthroplasty

☐ Yes ☐ No: Current hip fracture → Suggest unfractionated heparin 5000 units subcutaneous bid in anticipation of spinal anesthetic.

☐ Yes ☐ No: Active malignancy

☐ Yes ☐ No: Planned major abdominal/pelvic surgery (evaluate case by case)

☐ Yes ☐ No: Consider other risk factors: ☐Morbid obesity ☐Bed rest > 3 days
☐First degree relative with unprovoked VTE
☐Other: ____________________

☐ Yes ☐ No: Active bleeding or risk factors: ____________________

In-Hospital Prophylaxis ordered (See Physician Orders)

☐ Mechanical ☐ Pharmacologic ☐ None

________________________ ________________________
Date/Time Practitioner's Signature

VENOUS THROMBOEMBOLISM PROPHYLAXIS OPTIONS

1. Mechanical options include graduated compression stockings or intermittent pneumatic compression devices. These are not recommended as the sole treatment for patients who are high-risk for VTE unless there is a high risk of bleeding. Mechanical options can be added to pharmacologic options for high-risk patients. Risks of mechanical VTE prophylaxis include skin breakdown or ulcer development, particularly in stroke patients.

2. Pharmacologic options include enoxaparin or unfractionated heparin. Patients at high risk of bleeding should generally not receive heparin. In patients with significant renal impairment or expected to require spinal/epidural anesthesia (e.g. hip fracture), unfractionated heparin is preferred over enoxaparin (shorter duration and reversible). Suggested doses:

	Normal weight	Obese	Underweight
Enoxaparin	40 mg subcutaneous daily	30 mg subcutaneous bid	30 mg subcutaneous daily
Unfractionated heparin	5000 units subcutaneous bid	5000 units subcutaneous tid	3000 units subcutaneous bid
