# Supplementary material for: Venous thromboembolism prophylaxis may cause more harm than benefit: an evidence-based analysis of Canadian and international guidelines
Source: Thromb J. 2018 Oct 10;16:25. doi: 10.1186/s12959-018-0180-6 (PMC6178253; doi:10.1186/s12959-018-0180-6)
Supplement: Supplementary file 2 — 'Opt-out' hospital VTE screening tool: treats almost all hospitalized patients. (PDF 252 kb) [file 12959_2018_180_MOESM2_ESM.pdf]

PREVENTION OF VENOUS THROMBOEMBOLISM (VTE) SHOULD BE CONSIDERED FOR ALL PATIENTS ADMITTED TO ACUTE CARE

STEP 1: Is thromboprophylaxis NOT INDICATED?

Reasons
- Patient fully mobile
- Length of stay of 1-2 days

→

Actions
- No routine prophylaxis
- Reassess daily

STEP 2: Is anticoagulant thromboprophylaxis CONTRAINDICATED?

Reasons
- Active bleeding
- High risk of bleeding

→

Actions
- Bilateral TED stockings
- Reassess daily

STEP 3: PROVIDE THROMBOPROPHYLAXIS

For almost all patients, the recommended thromboprophylaxis is: enoxaparin (Lovenox®) 40 mg SC once daily*

***Exceptions**
- reduce dose to 30 mg SC once daily for weight < 40 kg or CrCl < 30 mL/min
- for patients with epidural catheters, give the dose qAM to facilitate catheter removal
- for selected patients (e.g. high risk trauma, obesity), enoxaparin 30 mg to 40 mg SC **BID** should be considered

Risk Factors for VTE

- Major surgery
- Trauma or leg injury
- Active cancer and its treatment
- Hypercoagulable states
- Immobilization, bedrest
- Acute medical illness
- Stroke
- Heart failure
- Previous history of VTE
- Family history of VTE
- Central venous catheter
- Pregnancy, postpartum
- Birth control pill, estrogen use replacement therapy
- Severe obesity
- Increasing age

Sunnybrook
HEALTH SCIENCES CENTRE

PR XXXXX (2010/08/10)
