# Supplementary material for: Venous thromboembolism prophylaxis may cause more harm than benefit: an evidence-based analysis of Canadian and international guidelines
Source: Thromb J. 2018 Oct 10;16:25. doi: 10.1186/s12959-018-0180-6 (PMC6178253; doi:10.1186/s12959-018-0180-6)
Supplement: Supplementary file 3 — Complex liberal hospital VTE risk-scoring tool: treats most hospitalized patients. (PDF 130 kb) [file 12959_2018_180_MOESM3_ESM.pdf]

BC WOMEN'S HOSPITAL+ HEALTH CENTRE
An agency of the Provincial Health Services Authority

Thromboprophylaxis for Venous Thromboembolism (VTE)

DATE _____/_____/_______ TIME _________
DD MM YYYY

WEIGHT:_________ KG HEIGHT______________CM

☐ ALLERGY CAUTION sheet reviewed

Patient Care

☐ Postpartum VTE Risk Assessment on Postpartum Orders Completed

- **Delivery Date: ____________________ Time:____________________**

Mechanical Prophylaxis:

INTERMEDIATE and HIGH RISK:

☐ Sequential Compression Devices (SCD)

- **Mobilize early and avoid dehydration**

Medications

Pharmacologic Prophylaxis:

☐ dalteparin 5000 unit subcutaneously once daily

- **Individualized dose:**

☐ dalteparin 7500 unit subcutaneously (use current BMI equal to or greater than 40) once daily

☐ dalteparin 2500 unit subcutaneously (for eGFR less than 30mL/min or weight equal to or less than 40 kg) once daily

- **DO NOT administer LMWH within 2 hours of epidural catheter removal**
- **See contraindications to low molecular weight heparin (LMWH) on reverse side**

Intermediate Risk:

Frequency

☐ Standard 6 hours post-delivery. Date: __________ Time: __________

OR

☐ Delay initial dose until Date: ___________ Time: _____________

- **THEN give dose once, daily (minimum of 24 hours after initial dose)**

☐ 1000 hours ***OR*** ☐ 2200 hours

- **Start Date: __________________**
- **Review for need for further prophylaxis after 72 hours if still in hospital**

High Risk:

- **Individual orders are based on antepartum consultation letter for pharmacologic prophylaxis recommendations (write order on blank prescriber's orders)**

At Discharge

☐ For Intermediate Risk: Discontinue prophylaxis at discharge

☐ For High Risk: Continue pharmacologic treatment for 6 weeks (prescription)

Signature:____ Print Name:_____________________________________

College ID:_______________________________________ Pager:____

BC WOMEN'S HOSPITAL & HEALTH CENTRE
An agency of the Provincial Health Services Authority

CHILDREN'S & WOMEN'S HEALTH CENTRE OF BRITISH COLUMBIA
AN AGENCY OF THE PROVINCIAL HEALTH SERVICES AUTHORITY

BC Women's Prescriber's Orders
Postpartum - Cesarean Births

Allergy ________________________

DATE dd/MON/yyyy	TIME hh:mm	WEIGHT kilograms (kg)	HEIGHT centimetres

Pharmacy Use Only	Checkmark All Tick Boxes That Apply.	Noted By RN/ UC
	**1. Postpartum Admission**<br>▪ Activity as tolerated, and sips to diet as tolerated<br>▪ Remove foley after 12 hours post – operative<br>▪ Remove staples post-operative day ☐ 2 ☐ 3 Other __________<br>▪ Intravenous (IV) D5W normal saline, infuse at 80 millilitres (mL)/ hour	

Medication	Dose	Route	Frequency	Indication
**2. Administered by Registered Nurse (RN)**				
☐ Oxytocin	______ units	IV	once in first litre	for bleeding
▪ Discontinue IV if bowel sounds are present and drinking well<br>▪ If IV needed for medications, change to saline lock				
☐ Glycerin suppository	one	per rectum	once daily as needed (prn)	constipation/gas
☐ Magnolax	30 mL	orally	once daily prn	constipation/gas
☐ Phosphate enema (Fleet)	one	per rectum	once prn	constipation
Rh negative (-) maternal blood group:<br>*(Check consent for Rh immune globulin on chart)*<br>☐ Rh immune globulin	120 micrograms	intramuscularly	once	If newborn is Rh positive or ☐ unknown
☐ MMR Vaccine (Measles-Mumps-Rubella)	0.5 mL	subcutaneously	once	Rubella non-immune
**3. Woman's Self - Administered Medications at RNs Discretion or Administered by RN. Or ☐ Do not self - administer medications.**				
☐ Docusate	100 milligrams	orally	twice daily prn	stool softener
☐ Anusol HC tube or substitute	one	apply rectally	twice daily & after bowel movement prn	hemorrhoids
**Additional Medications to Start 48 hours Post - operatively**				
**4. Medication administered by RN only.**				
☐ Hydromorphone	2 - 4 milligrams	orally	every 4 hours prn	break-through pain
**5. Woman's Self - Administered Medications at RNs Discretion or Administered by RN. Or ☐ Do not self - administer medications.**				
☐ Acetaminophen	325 - 650 milligrams	orally	every 4 - 6 hours prn<br>maximum 4 grams in 24 hours	pain
☐ Ibuprofen	400 milligrams	orally	every 4 - 6 hours prn	pain

6. Venous Thromboembolism (VTE) Risk Assessment *(COMPLETE reverse side)*
☐ Low Risk ☐ Intermediate/ High Risk *(Obtain & complete Postpartum VTE Orders BCW210)*

7. Physician responsible for care postoperative day:
#1 ______________________________ #3 ______________________________
2 ______________________________ #4 ______________________________

Physician's Signature ______________________________ CPSID# ____________
Print name ______________________________ Pager Number ________________

☐ **Photocopy** and send to Pharmacy

POSTPARTUM RISK ASSESSMENT FOR VENOUS THROMBOEMBOLISM (VTE) PROPHYLAXIS

Following delivery the prescriber completes the Postpartum Risk Assessment.
(✓ Checkmark all tick boxes that apply.)

☐ **No Risk Factors**

Pre-existing
- ☐ Age greater than or equal to 35 years
- ☐ Gross varicose veins
- ☐ Obesity, pre-pregnant BMI greater than or equal to 30 kg/m^2
- ☐ Smoker (current)

Postpartum
- ☐ Anticipated immobility
- ☐ Elective cesarean section
- ☐ Forceps with vaginal trauma
- ☐ PPH greater than 1 litre or blood transfusion
- ☐ Prolonged active labour (greater than 24 hours)
- ☐ Surgical procedure in postpartum

0 to 1 Risk Factor → **LOW RISK**

Equal to or greater than 2 Risk Factors → **INTERMEDIATE RISK**

- ☐ Antenatal immobility greater than 7 days
- ☐ Cesarean section in active labour
- ☐ Intravenous drug use
- ☐ Obesity, current BMI greater than 40 kg/m^2
- ☐ Medical co-morbidities including:
  - Acute medical illness/ sepsis
  - Autoimmune disease/ SLE
  - Cancer
  - Current active systemic infection
  - Heart or lung disease
  - Nephrotic syndrome
  - Pre-eclampsia

Any 1 Risk Factor → **INTERMEDIATE RISK**

- ☐ Previous DVT/ PE
- ☐ Treatment with therapeutic antenatal LMWH
- ☐ Asymptomatic thrombophilia, consult specialist

Any 1 Risk Factor → **HIGH RISK**

Key:
BMI = body mass index
DVT = deep vein thrombosis
kg = kilogram
LMWH = low molecular weight heparin
m = metre
PE = pulmonary edema
PPH = postpartum hemorrhage
SLE = Systemic lupus erythematosus

Refer to online version – Print copy may not be current – Discard after use
